# Epstein-Barr Virus Reactivation Causing Cholestatic Hepatitis

**DOI:** 10.7759/cureus.24552

**Published:** 2022-04-28

**Authors:** Teresa Da Cunha, Sheena Mago, Roopjeet K Bath

**Affiliations:** 1 Internal Medicine, University of Connecticut Health, Farmington, USA; 2 Gastroenterology, Allegheny Health Network, Pittsburgh, USA; 3 Gastroenterology and Hepatology, University of Connecticut Health, Farmington, USA

**Keywords:** cholestatic jaundice, cholestatic liver injury, viral hepatitis, hepatitis, epstein-barr virus

## Abstract

Infection with Epstein-Barr virus (EBV) is common and associated with a high seroprevalence. It is often asymptomatic, but infectious mononucleosis (IM) is the clinical hallmark of this disease especially among teens. Hepatic involvement during primary EBV infection often results in mild self-resolving elevation of liver enzymes, typically in association with IM. However, cholestatic hepatitis might sporadically occur. EBV reactivation is rare, especially among immunocompetent patients. Moreover, reactivation of EBV causing isolated cholestatic hepatitis is extremely rare and only reported in patients who are immunocompromised. Here we present a unique case of EBV reactivation causing cholestatic hepatitis in an otherwise healthy and immunocompetent female and we review the epidemiology, clinical presentation, diagnosis, and treatment of EBV induced cholestatic hepatitis.

## Introduction

Epstein-Barr virus (EBV) is a member of the herpes virus family with a very high seroprevalence. EBV transmission mostly occurs through saliva in which infected B cells are present [1]. The virus plays a role in a wide range of diseases including lymphoproliferative disorders and some lymphomas (Burkitt lymphoma, Hodgkin, and diffuse large B-cell lymphomas) but usually, infection with this virus is completely asymptomatic [2]. When symptomatic, infectious mononucleosis (IM) is the most common presentation and teenagers are at higher risk [3]. Hepatic involvement during an active primary EBV infection often results in mild, self-resolving elevation of transaminases which is typically subclinical and associated with IM [4]. Nonetheless, there are sporadic reports of EBV-induced cholestatic hepatitis [5].

Chronic EBV infection can also cause hepatitis [6]. However, reactivation of EBV is rare and immunocompromised patients are at higher risk [7,8]. We present a unique case of isolated cholestatic hepatitis caused by reactivation of EBV in an immunocompetent patient and we review the epidemiology, clinical presentation, diagnosis, and treatment of EBV induced cholestatic hepatitis.

This article was previously presented as a meeting abstract at the ACG conference 2021.

## Case presentation

A 19-year-old woman with a history of self-resolved EBV IM one year prior, presented to the hospital complaining of dark urine and yellow discoloration of the skin. Otherwise, she was asymptomatic and denied any fever, sore throat, joint pain, abdominal pain, nausea, vomiting, weight changes, or night sweats. She denied a history of smoking, illicit/recreational drug use, alcohol use, recent travel, or any sick contacts. Her medications included loratadine for seasonal allergies and an oral contraceptive pill. She was not taking any acetaminophen or herbal supplements. The family history was unrevealing.

**Figure 1:**
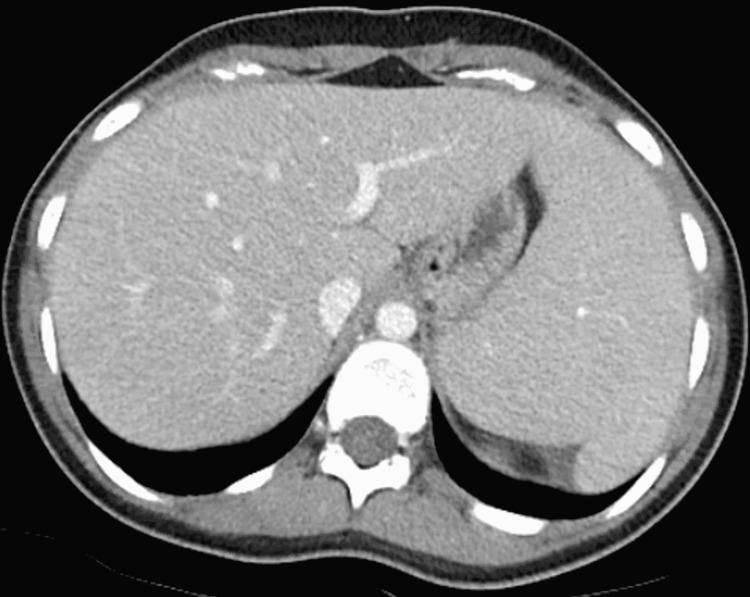
Computed tomography of the abdomen revealing splenomegaly but no other relevant findings.

**Figure 2:**
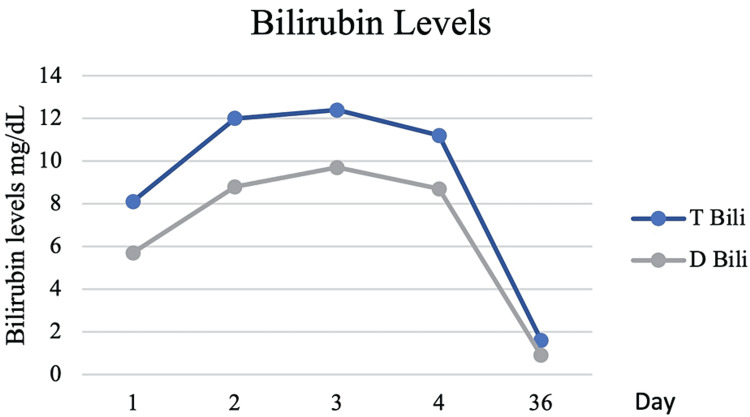
Total and direct bilirubin levels throughout hospitalization and at follow-up.

**Figure 3:**
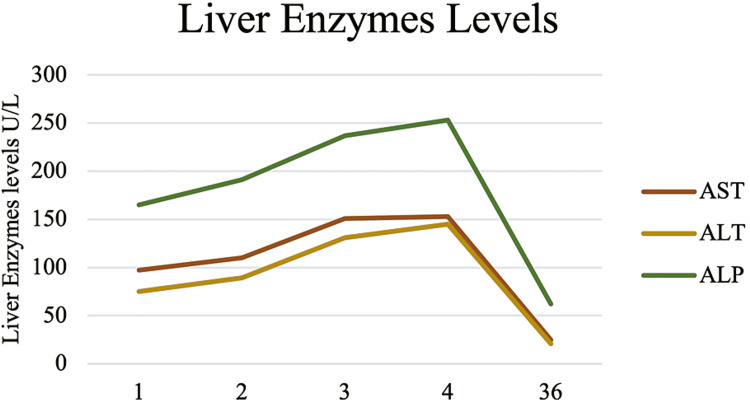
Liver transaminases levels throughout hospitalization and at follow-up.

On exam, she was noted to have jaundice but no lymphadenopathy, her abdomen was soft, non-tender, and without organomegaly on palpation. The initial laboratory blood results were notable for aspartate aminotransferase (AST) 97 U/L, alanine aminotransferase (ALT) 75 U/L, alkaline phosphatase (ALP) 165 U/L, total bilirubin (T-BILI) 8.1 U/L, direct bilirubin (D-BILI) 5.7 mg/dL. Computed tomography of the abdomen noted mild splenomegaly but was otherwise unremarkable (Figure 1). Further workup revealed an anti-smooth muscle antibody titer of 1:40, but otherwise negative/ normal anti-nuclear antibody, IgG level, anti-mitochondrial antibody, hepatitis C virus antibody, hepatitis B virus, and hepatitis A virus serologies, cytomegalovirus (CMV) polymerase chain reaction, tick-borne serologies, SARS CoV-2 PCR, alpha-1 antitrypsin, transferrin saturation, and ceruloplasmin levels. On the second day of hospitalization, the T-BILI peaked at 12.4 mg/dL, AST 151 U/L, ALT 131 U/L, ALP 237 U/L (Figures 2, 3).

 

At this time, EBV serologies were positive (EBV viral capsid antigen IgG > 750.0 U/mL, EBV viral capsid antigen IgM > 160.0 U/mL), with an EBV viral load of 23,500 copy/mL and negative monospot test. For this reason, a diagnosis of hepatitis due to EBV reactivation was established. Without any intervention, the liver chemistries improved, and she was discharged home on the fourth day. One month later, the liver tests had normalized, and she was completely asymptomatic.

## Discussion

Infection with EBV is common and studies have shown high seropositive rates, especially in developing countries, ranging 80%-100% in children [9]. In developed countries, its incidence tends to be lower, and the infection seems to occur at a later age. In the study of Chan et al., the estimated seroprevalence of EBV in the US is around 54.1% in children 6-8 years old and 82.9% in those 18 to 19 years old. Moreover, statistically significant differences in prevalence are associated with socioeconomic status; children from families with lower income are at higher risk of infection [10]. The development of IM usually occurs in younger adults and the estimated incidence in the U.S. is 500 cases per 100,000 per year [3]. Although mild asymptomatic elevation of transaminases during primary EBV infection is relatively common, EBV causing cholestatic hepatitis is uncommon with few isolated case reports in the literature. A large retrospective study done in the UK by Vine et al. found that EBV-causing hepatitis accounted for 0.85% (17 of 1,995 cases) of hepatitis cases with a median age of 40 years, of the 17 cases, there were 10 males and seven females [5]. It is important to note that not all 1,995 cases were tested for EBV given that once a separate diagnosis was established, EBV testing was not required. Isolated hepatitis due to EBV reactivation is extremely rare, especially among immunocompetent hosts.

**Figure 4:**
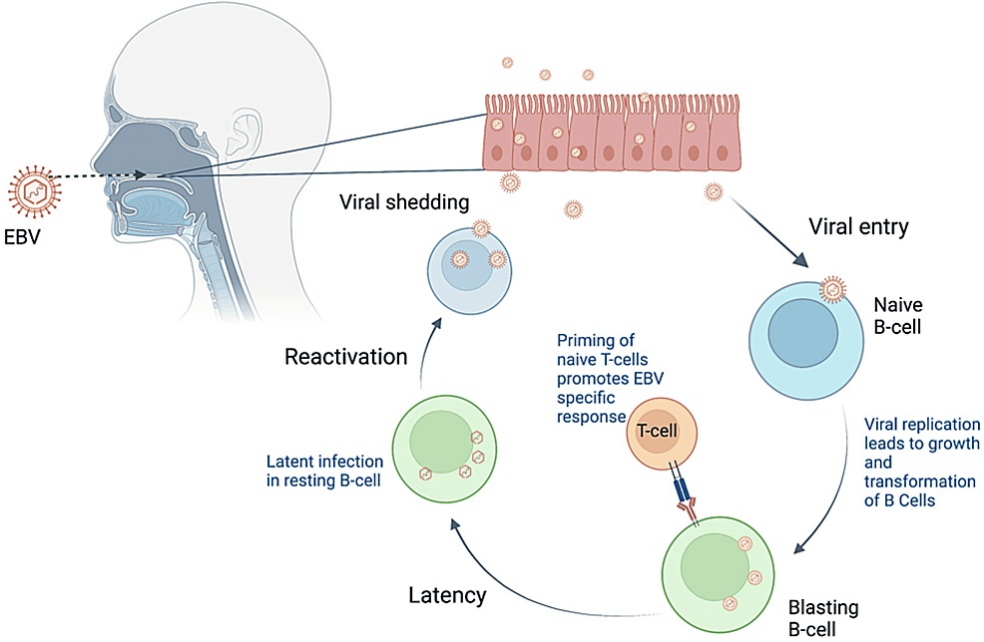
Schematic representation of infection and reactivation by Epstein-Barr virus. Image produced using Biorender. Image credits: Teresa Da Cunha, MD.

EBV is mainly transmitted through salivary transfer by EBV-infected B cells. Similar to other herpes viruses, EBV has the ability to remain in a latent state inside B cells (immunoblastic B cells, memory B cells, resting non-immunogenic B cells) by means of modulation of apoptotic signals and cytokines [11]. Under specific circumstances, reactivation of the virus can occur, leading to disease (Figure 4). Immunocompromised patients are at higher risk for EBV reactivation, but rarely reactivation can also occur in immunocompetent individuals. Situations that impair effective cellular immune response, including any type of psychologic stress, might trigger EBV reactivation in immunocompetent individuals [12,13]. Reactivation of EBV has also been seen in association with autoimmune diseases including systemic lupus erythematosus, rheumatoid arthritis, and multiple sclerosis [13].

Similar to CMV, liver injury during EBV infection is thought to result mainly from indirect cytopathogenicity given the abundance of lymphocytes present on histological examination and the rarity of EBV-infected B cells [14,15]. The exact mechanisms leading to hepatitis from EBV reactivation and chronic infection however are not well established.

Primary infection during childhood usually does not cause any symptoms, however in adolescence, it can lead to IM in about 30%-50% of cases, which is characterized by symptoms including fatigue, tonsillitis and fever [10]. Immunocompetent patients can also present with weight loss and flu-like symptoms. Elevations of aminotransferases with or without bilirubin elevation are expected if there is liver injury during EBV infection. Mild elevations of transaminases during IM are common; however, cholestatic hepatitis is uncommon and usually associated with a higher degree of hepatocellular injury [16]. Leonardsson et al. described 190 patients that presented to the hospital due to acute EBV infection; 82% (n=156) had elevated liver enzymes and 15% (n=24) had cholestatic hepatitis causing jaundice. The higher rate of jaundice in this study, compared to prior studies reporting around 5%, is likely because these patients were sick enough to present to the hospital [4,17]. The median age of these patients was 17 years and 98% of them had some symptomatology of IM. Moreover, hepatomegaly and splenomegaly were identified in 11% and 12% of cases, respectively. In the study by Vine et al., of the 17 patients with EBV hepatitis, only two of them had the classic symptoms of IM. The discrepancy in findings is likely due to differences in the initial selection of patients. Leonardsson et al. selection was done from a large group of patients with EBV who also had hepatitis, whereas Vine et al. selected the EBV-related hepatitis cases from a large group of patients with hepatitis [4,5].

The differences in the study population may also explain the differences in liver function test profiles. In the study by Leonardsson et al. the average level for ALT, AST, ALP, and T-BILI were 187, 137, 214 and 1.64 mg/dL, respectively [4]. Those from Vine et al. had a significantly higher level, ALT 395 U/L, ALP 345, T-BILI 4.33 mg/dL [11]. Interestingly, our patient had a significantly higher degree of cholestasis with a peak T-BILI of 12.4 but mild hepatocellular injury. In contrast, the other two cases of hepatitis from EBV reactivation had a moderate to severe elevation of transaminases and significant cholestasis [18,19].

Given hepatitis can be caused by a multitude of disease states, a thorough workup is necessary to exclude other causes including evaluation for hepatitis A, B and C, CMV, HSV, autoimmune hepatitis, and drug-induced liver injury. Depending on chronicity, evaluation with iron studies, ceruloplasmin, and alpha-1 antitrypsin levels may also be indicated. The American College of Gastroenterology only recommends evaluation for EBV serology when there are moderate (5-15x higher than the upper limit of normal levels), severe and massive elevations of ALT and/or AST, but mild elevations are often seen, including in our case [4]. When other etiologies have been ruled out and the EBV serologies are positive the diagnosis can be established, precluding the need for liver biopsy.

EBV reactivation causing hepatitis or mild elevation of transaminases is rare and immunocompromised individuals are at higher risk, specifically those on immunomodulators [7,20]. Cases of acute liver failure resulting in death have been reported due to EBV reactivation in immunocompromised patients [18]. Although EBV-induced fulminant hepatitis has occurred, the prognosis of EBV hepatitis during primary EBV infection is usually excellent and, in most cases complete recovery is achieved with symptomatic treatment only [4,5]. Due to the lack of data, it is difficult to determine whether EBV hepatitis caused by EBV reactivation has the same prognosis. From the few reports, the one with fatal hepatitis was initially misdiagnosed with rheumatoid arthritis due to multiple joint pain and was started on a high dose of prednisone which could have negatively impacted the outcome [18]. One other patient was initially misdiagnosed with natural killer cell/T-cell lymphoma and was started on rituximab. He required treatment with valganciclovir due to worsening liver injury but subsequently achieved full recovery [19]. In our case, full recovery was attained without intervention. The fact that our patient was immunocompetent and otherwise healthy likely contributed to the good outcome.

## Conclusions

EBV reactivation causing cholestatic liver injury is extremely rare in immunocompetent individuals, and to our knowledge, this is the first report of an otherwise healthy patient not on any type of immunosuppressive therapy. The fact that the hepatitis was caused by the reactivation of the virus instead of a primary infection may be the reason for the lack of other symptoms. It is unclear whether EBV reactivation increases the risk for hepatitis. The diagnosis can be made without a liver biopsy if thorough work up to evaluate hepatitis is negative and the EBV serologies are positive. The disease can be self-limited; hence, the use of antivirals is controversial, and thus there is no clear indication. Our case demonstrates that EBV reactivation presenting without the “classic" IM symptoms may be under-recognized as a cause of cholestatic liver injury.
